# The ecology of medical care on the westernmost remote island, Yonaguni Island, Japan: A cross-sectional study

**DOI:** 10.1371/journal.pone.0199871

**Published:** 2018-06-28

**Authors:** Hirofumi Namiki, Tadashi Kobayashi

**Affiliations:** 1 Yonaguni Municipal Clinic, Japan Association for Development of Community Medicine, Okinawa, Japan; 2 Department of General Medicine, Hirosaki University School of Medicine & Hospital, Aomori-ken, Japan; New York City Department of Health and Mental Hygiene, UNITED STATES

## Abstract

Yonaguni Island is a remote and isolated westernmost island in Japan, which is the fastest aging country in the world. This study evaluated the current status of medical supply-and-demand on the island and compared these results with previous surveys carried out in rural parts of Japan. This was a retrospective cohort study conducted at the Yonaguni Municipal Clinic, the only medical facility in Yonaguni Island. The participants were patients who visited the clinic over one year, between July 2015 and June 2016. We calculated the rate per 1,000 persons per month of clinic visits, referrals to off-island medical facilities (e.g., hospitals and specialist clinics), referrals to off-island emergency departments, off-island hospitalizations after referral, home visits, and overnight observations at the clinic. In total, 6,197 patients (males, 46.3%) visited the clinic. The rate of clinic visits per 1,000 persons per month was 516.4 (Standard deviation [SD] 28.1, 95% confidence interval [CI]: 500.5–532.3). The rate per 1,000 persons per month was 14.0 (SD 3.9, 95% CI: 11.8–16.2) for off-island referrals, 3.8 (SD 2.1, 95% CI: 2.6–5.0) for referrals to emergency departments, 4.8 (SD 2.6, 95% CI: 3.3–6.2) for hospitalizations, and 3.2 (SD 1.7, 95% CI: 2.2–4.1) for home visits. The rate of clinic visits was higher in Yonaguni Island than in other rural areas, although the rate of off-island referrals was lower. There were no significant differences between the number of referrals to emergency departments, hospitalizations, and home visits in Yonaguni Island, in comparison to other studies. Our study showed that patients presenting with emergencies had similar rates of healthcare-seeking behavior to those reported in previous studies in Japan; however, the referral rate was lower. We assessed the ecology of medical care in this district by evaluating patient behavior on an isolated island where access to medical care is geographically limited.

## Introduction

The aging of society and the resulting increase in medical expenses are serious concerns for developed and developing countries, yet to be faced with aging [1,2]. Japan has received much international attention for maintenance of good health in the population, by way of free access to medical care and low medical fees under the universal health insurance coverage [3]. However, the burden on healthcare providers and public healthcare costs are expected to increase rapidly since Japan is the fastest aging country in the world [4,5]. In Japanese remote and isolated islands, there are issues regarding rapid population aging, estimations of the actual medical supply and demand, and the future reconstruction needed in consideration of the geographical limitations.

White et al. proposed a framework for medical supply-and-demand in their article titled, “The Ecology of Medical Care” [6]. Based on their framework, surveys on the actual ecology of medical care have also been conducted in Japan, focusing on urban areas [7,8]. In contrast, fewer studies have reported on the ecology of medical care in remote and isolated islands [9].

Our study surveyed the actual status of medical supply-and-demand, in relation to all the patients who visited the Yonaguni Municipal Clinic, the only medical facility in Yonaguni Island, located in the westernmost region in Japan, in order to assess the quality of healthcare, and adjust the quantity of supply-and-demand for medical services in a rural area where medical access is geographically limited. Our results have been compared with those of two previous surveys carried out in rural Japan; Fukui et al.(2017) carried out the most representative report on the ecology of medical care in Japan [8], and Kaneko et al.(2017) researched a different remote island in Okinawa, Japan [9].

## Materials and methods

This study was approved by the Ethics Committees of the Japan Association for Development of Community Medicine (ID: 16–02) and the Hirosaki University School of Medicine & Hospital (ID: 2016–1113).

As of June 2016, the Yonaguni Municipal Clinic is the only medical facility in Yonaguni Island, a remote island in Okinawa Prefecture, Japan (area, 28.9 km^2^; population, 1,696 (males, 812); population aging rate >65 years, 18.5%; proportion of young people <15 years, 12.5%). The island is located 510 km from the main island of Okinawa (90 minutes by airplane: one-way fare approximately 13,300 Japanese yen). The clinic had six staff members, including one physician (one of the present authors, HN), three nurses, and two clerks. No inpatient treatment was available, but overnight observation was available in the outpatient treatment room. A 24-hour emergency call system was in place (approximately 50 emergency calls or patient consultations per month during the nighttime). The available medical equipment included a blood-test analyzer (e.g., blood count and general biochemical examination), a microscope for examining bacteria (e.g., Gram stain), an electrocardiography system, roentgenographic equipment, diagnostic ultrasound equipment, and computed tomography (CT) equipment. There is no facility on the island of Yonaguni that provides alternative or complementary medicine.

The participants in our study were all the patients who had visited the Yonaguni Municipal Clinic between July 2015 and June 2016. The following evaluation items were retrospectively counted: the rate of clinic visits per 1,000 persons per month, the rate of referrals to off-island medical facilities (e.g., hospitals and specialist clinics), the rate of referrals to off-island emergency departments, the rate of off-island hospitalizations after referral, the rate of home visits in Yonaguni Island, and the rate of overnight observations at the clinic. The unit of analysis was one per -month, indicating receipt of services in a healthcare setting at least once in a month. Our Yonaguni survey was evaluated using a standard deviation (SD) because it represented a full-number survey. We directly compared the rate of clinic visits per 1,000 persons per month to those recorded in other Japanese studies analyzing visits, referrals, hospitalizations, emergencies, and home visits, using a 95% confidence interval (CI).

Using the software Easy R [10], we performed descriptive analyses to evaluate the healthcare-seeking behavior per 1,000 inhabitants over a 1-month period, based on the actual number of visits. We calculated the 95% CI for event rates with either a normal distribution (≥10 events) or a Poisson distribution (<10 events).

## Results

**Fig 1:**
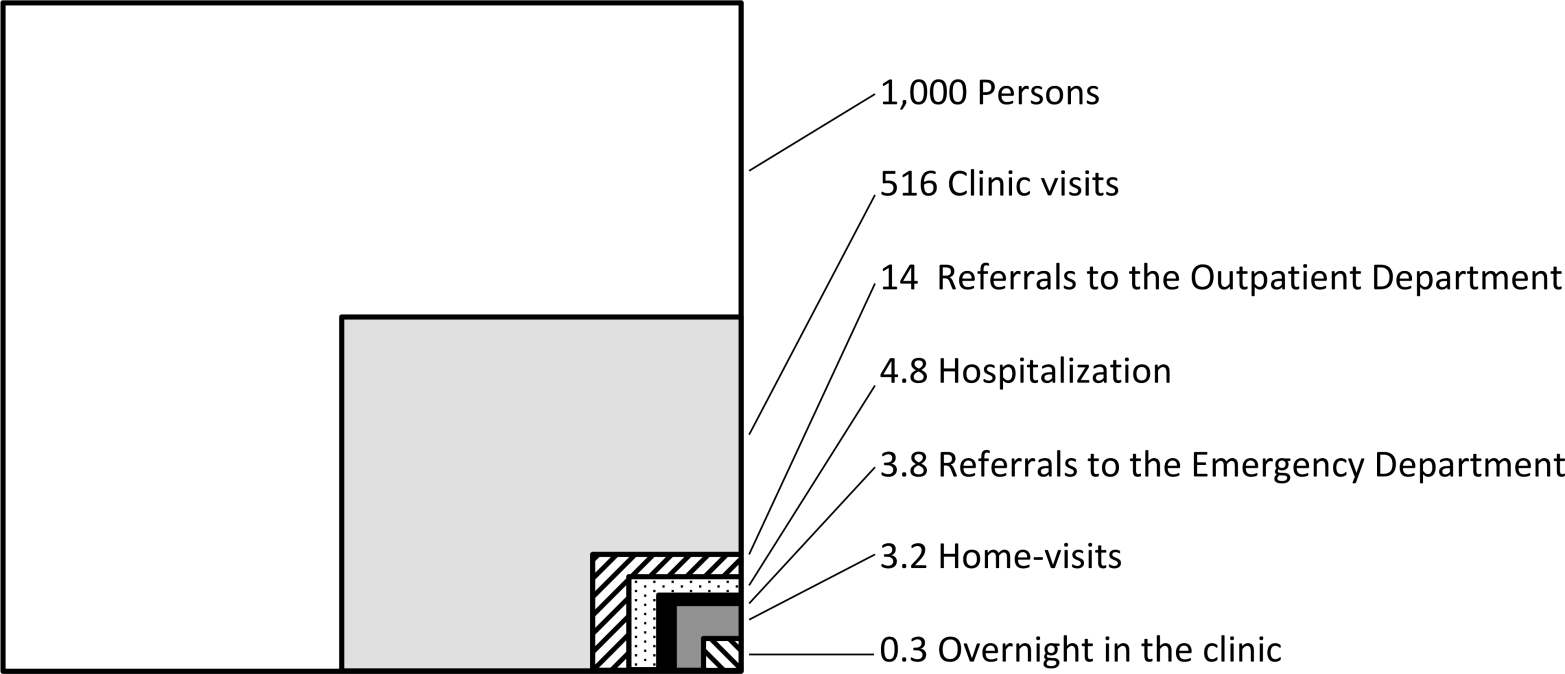
Medical ecology in the present study of Yonaguni island residents: Rates of healthcare-seeking behaviors per 1,000 persons per month.

In total, 6,197 patients (males, 46.3%) visited the clinic. Of these, 827 (males, 50.2%) were aged <15 years; 3,103 (males, 49.6%) were aged 15–65 years; and 2,267 (males, 40.5%) were aged ≥65 years. The rate of clinic visits per 1,000 persons per month was 516.4 (SD 28.1, 95% CI: 500.5–532.3). The rate of referrals to the outpatient departments of other medical facilities was 14.0 (SD 3.9, 95% CI: 11.8–16.2). The rate of off-island referrals to the emergency departments of other medical facilities was 3.8 (SD 2.1, 95% CI: 2.6–5.0). The rate of hospitalizations at other medical facilities after referral was 4.8 (SD 2.6, 95% CI: 3.3–6.2). The rate of home visits in Yonaguni Island was 3.2 (SD 1.7, 95% CI: 2.2–4.1). The rate of overnight observations at the clinic was 0.3 (SD 0.5, 95% CI: 0.1–0.9). The details and summary are shown in Fig 1 and Table 1. The comparison between our study and other areas in Japan is shown in Table 2.

**Table 1:** Rates of healthcare-seeking behavior per 1,000 persons per month in Yonaguni island.

	Visits (SD)	Referrals to the OD (SD)	Referrals to the ED (SD)	Hospitalization (SD)	Home visits (SD)	Overnight (SD)
Overall	516.4 (28.1)	14.0 (3.9)	3.8 (2.1)	4.8 (2.6)	3.2 (1.7)	0.3 (0.5)
Ages						
<15	68.9 (10.8)	0.8 (0.9)	0.3 (0.4)	0.3 (0.4)	2.4 (1.3)	0 (0)
15–70	273.4 (19.5)	8.8 (2.0)	1.8 (1.3)	2.4 (1.5)	0.5 (0.6)	0.2 (0.4)
70>	174.1 (16.1)	4.3 (2.3)	1.8 (1.3)	2.1 (1.8)	0.3 (0.4)	0.2 (0.4)
Sex						
males	239.3 (16.9)	7.8 (3.1)	2.4 (1.5)	2.8 (1.8)	1.2 (0.8)	0.2 (0.4)
females	277.1 (17.5)	6.2 (2.4)	1.4 (1.0)	1.9 (0.9)	2.0 (1.1)	0.2 (0.4)

Table abbreviation: SD, standard deviation; OD, Outpatient Department; ED, Emergency Department.

**Table 2:** Comparison of rates of healthcare-seeking behaviors per 1,000 persons per month between our study and previous studies in Japan.

	Fukui et al. (2017),^[^^8^^]^ rural area, Japan (95% CI)	Kaneko et al. (2017),^[^^9^^]^ Iheya, Okinawa (95% CI)	Our study of Yonaguni, Okinawa (95% CI)
Visits to primary care clinics	224.0 (178.0–275.0)	360.4 (351.0–369.7)	516.4 (500.5–532.3)
Visits to the OD of other medical facilities*	69.0 (43.0–104.0)	18.4 (16.3–20.5)	14.0 (11.8–16.2)
Visits to the ED of other medical facilities	3.0 (<1–18.0)	4.1 (3.1–5.1)	3.8 (2.6–5.0)
Hospitalization†	7.0 (1.0–24.0)	3.6 (2.6–4.6)	4.8 (3.3–6.2)

* Fukui et al. (2017) included data on both referral and non-referral visits. Kaneko et al. (2017) and the present study included only referral visits.

† Fukui et al. (2017) included data on both referral and non-referral hospitalizations at all medical facilities. Kaneko et al. (2017) and the present study included only referral hospitalizations at off-island medical facilities.

Abbreviations: 95% CI, 95% confidence interval; Outpatient Department; ED, Emergency Department.

## Discussion

In this study, we assessed the ecology of medical care in Yonaguni Island, an isolated island where access to other medical facilities is geographically limited, by investigating the healthcare-seeking behaviors of all patients, and evaluated the comparison to other rural areas.

The rate of visits to primary care clinics was higher in our study than that reported by Fukui et al. (2017) [8] who studied urban and rural areas in Japan. The discrepancy was attributable to the isolated nature of the island, including limited access to other medical facilities, difficulty purchasing over-the-counter drugs (owing to the absence of pharmacies), and the extreme scarcity of medical services, including alternative medicine, other than the clinic [11, 12]. The rate of visits to primary care clinics in our study was also higher than that reported in the study by Kaneko et al. (2017) [9]. This was because the island they studied was closer to the main island of Okinawa (41.1 km away), whereas Yonaguni Island is located 507.8 km away. Therefore, the factors thought to underlying the increased number of visits to the clinic were poor access to advanced medical facilities, lack of medical services other than the clinic, and the adequate allocation of healthcare resources through the gatekeeping role of the clinic.

The rate of visits to the outpatient department of other medical facilities in our study was lower than that reported by Fukui et al. (2017) [8]. This was due to reasons such as difficulty in visiting advanced medical facilities due to time and economic constraints, and the function of the only clinic in the island as a gatekeeper, which enabled adequate allocation of medical resources. The rate of visits to the outpatient departments of other medical facilities in our study was lower than that reported by Kaneko et al. (2017) [9]; this finding may be explained by high diagnostic accuracy in emergency cases, due to the availability of CT equipment (including remote reading) at the Yonaguni Municipal Clinic, despite the difference in access to advanced medical facilities on the main island of Okinawa.

There were no significant differences in the rates of referral to emergency departments of off-island medical facilities and hospitalizations between our study and previous studies [8,9]. This could be explained by the fact that the incidence of diseases presenting as emergencies and/or requiring hospitalization did not differ from one district to another, and that there was ready access to advanced medical facilities in emergency cases.

The rate of home visits, 3.7 per 1,000 persons per month in Okinawa Prefecture (population size 1,423,000 in September 2016; population aging rate >65 years, 19.4%) in October 2014, was the lowest in Japan [13]. The rate of home visits, 3.2 (95% CI: 2.2–4.1) per 1,000 persons per month in our study of Yonaguni Island (population size 1,696 in June 2016; population aging rate >65 years, 18.5%) was about the same as in Okinawa Prefecture.

The rate of overnight observations at the clinic per 1,000 persons per month in our study was 0.3 (95% CI: 0.1–0.9). In some countries, overnight observation is provided as a service for patients who may require inpatient care in the emergency department or another area of the hospital, but not in facilities are primary care centers. We suggest that the provision of overnight observation may have helped to reduce, not only the number of hospitalizations [14], but also the burden on other medical facilities, and the time and economic constraints faced by patients. Healthcare in rural areas in Japan might be supported by specific and devoted efforts, including the overworking of healthcare staff, a problem for which Japan is world-famous [15].

We attempted to compare the findings of our study with those obtained in rural parts of other countries, including Australia [16], Belgium [17], China [18], Sweden [19], the U.S. [20] (S1 Table). However, the following factors made it impossible to apply the appropriate statistical methods: few raw data were disclosed in published studies (e.g., unavailability of data from rural areas, unseparated data for urban and rural areas); there were significant differences in the types of areas researched (e.g., metropolitan/non-metropolitan, rural/urban); there were substantial differences in healthcare systems and access to medical facilities (e.g., patients with or without private/public insurance, missing data from rural areas); there were also large differences of the adoption rate for alternative medicine worldwide, especially high rate in Asia, which affected healthcare-seeking behaviors (e.g., date with or without alternative medicine).

Isolated island societies are facing aging and shrinking populations. The following strategies are needed to develop sustainable and effective medical systems for the inhabitants of isolated islands: 1) establishment of an efficient referral system for patients requiring emergent treatment or advanced medical care [21, 22], 2) introduction of cost-effective medical interventions [6] to accommodate for more patients who require home visits and end-of-life care [4], and 3) enhancement of the effective utilization of mobile health services by using information and communication technology [23]. We hope that these strategies will be relevant to other countries that will soon be faced with aging populations.

### Limitations

The participants in our study were limited to only patients who visited the Yonaguni Municipal Clinic. Although almost all Yonaguni residents use the clinic, some live outside the island, despite being registered as residents because of their medical conditions (e.g., end-of-life care and dialysis treatment), and other reasons (e.g., seeking advanced medical care and seeking to see specialists off the island). Thus, the participants in our study do not represent all patients on the island.

## Conclusions

The rate of clinic visits was higher, and the number of referrals to off-island medical facilities was lower, than those found in previous studies. There was no significant difference between the present study and previous research in relation to the rate of hospitalizations and referrals to off-island emergency departments. This study has described the ecology of medical care in Yonaguni Island, an isolated island, where access to other medical facilities is geographically limited, by assessing the healthcare-seeking behaviors of all patients who visited the island’s single clinic.

## Conflicts of interest

None.

## Supporting information

S1 Table Comparison of rates of healthcare-seeking behaviors per 1,000 persons per month in the present study and in previous studies from Australia, Belgium, China, Sweden, and the U.S. *The Australian study [16] did not analyze urban and rural areas separately.
†In the Belgian study [17], the data included visits to both doctor’s offices and outpatient departments. Referrals were not clearly indicated.
‡In the Chinese study [18], the data included visits to both primary care clinics and hospital outpatient departments. The data of rural area and referrals were not included.
§In the Swedish study [19], referrals included hospital and university hospital outpatients, and hospitalizations included both hospital and university hospital inpatients.
||The U.S. study [20], collected both MSA and non-MSA data; rural referrals related to hospital outpatient department visits.
Abbreviations: non-MSA, non-metropolitan statistical area; OD, Outpatient Department; ED, Emergency Department; N/A, not available.
(PDF)

S1 File Minimal underlying data set. (PDF)

S2 File STROBE statement checklist. (PDF)

## References

[1] Green LA, Fryer GE Jr, Yawn BP, Lanier D, Dovey SM. The ecology of medical care revisited. N Engl J Med. 2001;344: 2021–5. doi: 10.1056/NEJM200106283442611 11430334 10.1056/NEJM200106283442611

[2] Johansen ME, Kircher SM, Huerta TR. Reexamining the Ecology of Medical Care. N Engl J Med 2016;374:495–6. 10.1056/NEJMc1506109 26840150

[3] Tamiya N, Noguchi H, Nishi A, Reich MR, Ikegami N, Hashimoto H, et al Population ageing and wellbeing: lessons from Japan’s long-term care insurance policy. Lancet. 2011;378(9797):1183–92. doi: 10.1016/S0140-6736(11)61176-8 21885099 10.1016/S0140-6736(11)61176-8

[4] Ikegami N, Yoo BK, Hashimoto H, Matsumoto M, Ogata H, Babazono A, et al Japanese universal health cover: evolution, achievement, and challenges. Lancet. 2011 9 24;378(9796):1106–15. doi: 10.1016/S0140-6736(11)60828-3 21885107 10.1016/S0140-6736(11)60828-3

[5] Hashimoto H, Ikegami N, Shibuya K, Izumida N, Noguchi H, Yasunaga H, et al Cost containment and quality of care in Japan: is there a trade-off? Lancet. 2011 9 24;378(9797):1174–82. doi: 10.1016/S0140-6736(11)60987-2 21885098 10.1016/S0140-6736(11)60987-2

[6] White KL, Williams TF, Greenberg BG. The ecology of medical care. N Engl J Med 1961;265:885–92. doi: 10.1056/NEJM196111022651805 14006536 10.1056/NEJM196111022651805

[7] Fukui T, Rhaman M, Takahashi O, Saito M, Shimbo T, Endo H, et al The ecology of medical care in Japan. JMAJ. 2005;48(4):163–7.

[8] Fukui T, Rahman M, Ohde S, Hoshio E, Kimura T, Urayama KU, et al Reassessing the ecology of medical care in Japan. J Community Health. 2017 3 31. Epub ahead of paper. 10.1007/s10900-017-0337-4 28364318

[9] Kaneko M, Matsushima M, Irving G. The ecology of medical care on an isolated island in Okinawa, Japan: a retrospective open cohort study. BMC Health Serv Res. 2017 1 14;17(1):37 doi: 10.1186/s12913-017-1979-8 28088204 10.1186/s12913-017-1979-8 PMC5237497

[10] Kanda Y. Investigation of the freely available easy-to-use software ‘EZR’ for medical statistics. Bone Marrow Transplant. 2013;48(3):452–8. doi: 10.1038/bmt.2012.244 23208313 10.1038/bmt.2012.244 PMC3590441

[11] Levinson W, Kallewaard M, Bhatia RS, Wolfson D, Shortt S, Kerr EA, et al 'Choosing Wisely': a growing international campaign. BMJ Qual Saf. 2015; 24(2):167–74. doi: 10.1136/bmjqs-2014-003821 25552584 10.1136/bmjqs-2014-003821

[12] Tokuda Y. Choosing Wisely International and Japan. General Medicine. 2015;16(2):61–2.

[13] E-stat. Portal site of official statics of Japan. http://www.e-stat.go.jp/SG1/estat/eStatTopPortalE.do

[14] Forrest CB. Primary care gatekeeping and referrals: effective filter or failed experiment? BMJ. 2003;326(7391):692–5. doi: 10.1136/bmj.326.7391.692 12663407 10.1136/bmj.326.7391.692 PMC152368

[15] Yoshitake K, Takashi O. Association of current work and sleep situations with excessive daytime sleepiness and medical incidents among Japanese physicians. J Clin Sleep Med. 2011;7(5):512–22. doi: 10.5664/JCSM.1322 22003348 10.5664/JCSM.1322 PMC3190852

[16] Sturmberg J, McDonnell G. How Modelling could Contribute to Reforming Primary Care—Tweaking “the Ecology of Medical Care” in Australia. AIMS Med Sci. 2016;3(3):298–311.

[17] Vo TL, Duchesnes C, Vögell O, Belche JL, Massart V, Giet D. The ecology of health care in a Belgian area. Acta Clin Belg. 2015;70(4):280–6. doi: 10.1179/0001551214Z.000000000137 25359713 10.1179/0001551214Z.000000000137

[18] Shao S, Zhao F, Wang J, Feng L, Lu X, Du J, et al The Ecology of Medical Care in Beijing. PLoS One. 2013 12 5;8(12):e82446 doi: 10.1371/journal.pone.0082446 24340029 10.1371/journal.pone.0082446 PMC3855438

[19] Ferreo A, Kristiansson PM. Ecology of medical care in a publicly funded health care system: A registry study in Sweden. Scand J Prim Health Care. 2011;29:187–92. doi: 10.3109/02813432.2011.585546 21707236 10.3109/02813432.2011.585546 PMC3347955

[20] Fryer GE Jr, Green LA, Dovey SM, Yawn BP, Phillips RL, Lanier D. Variation in the ecology of medical care. Ann Fam Med. 2003;1:81–9. doi: 10.1370/afm.52 15040437 10.1370/afm.52 PMC1466566

[21] Ministry of Health, Labor and Welfare, Japan. Patient’s Behavior Survey 2011. http://www.mhlw.go.jp/english/database/db-hss/pbs_2011.html

[22] Reich MR, Shibuya K. The Future of Japan's Health System—Sustaining Good Health with Equity at Low Cost. N Engl J Med. 2015;373(19):1793–7. doi: 10.1056/NEJMp1410676 26535509 10.1056/NEJMp1410676

[23] Miyata H, Ezoe S, Hori M, Inoue M, Oguro K, Okamoto T, et al Japan’s vision for health care in 2035. Lancet. 2015;385(9987):2549–50. doi: 10.1016/S0140-6736(15)61135-7 26122147 10.1016/S0140-6736(15)61135-7

