# Supplementary material for: The ecology of medical care on the westernmost remote island, Yonaguni Island, Japan: A cross-sectional study
Source: PLoS One. 2018 Jun 28;13(6):e0199871. doi: 10.1371/journal.pone.0199871 (PMC6023172; doi:10.1371/journal.pone.0199871)
Supplement: S1 Table — *The Australian study [16] did not analyze urban and rural areas separately. †In the Belgian study [17], the data included visits to both doctor’s offices and outpatient departments. Referrals were not clearly indicated. ‡In the Chinese study [18], the data included visits to both primary care clinics and hospital outpatient departments. The data of rural area and referrals were not included. §In the Swedish study [19], referrals included hospital and university hospital outpatients, and hospitalizations included both hospital and university hospital inpatients. ||The U.S. study [20], collected both MSA and non-MSA data; rural referrals related to hospital outpatient department visits. Abbreviations: non-MSA, non-metropolitan statistical area; OD, Outpatient Department; ED, Emergency Department; N/A, not available. (PDF) [file pone.0199871.s001.pdf]

	Australia*, urban/rural area	Belgium†, urban/rural area	China‡, urban area	Sweden§, rural area	U.S. ‖, non-MSA	Our study, Yonaguni, Japan
Visits to the OD	255	609	173	88	213.2	516.4
Referrals	42	N/A	N/A	44.9	25.7	14
Visits to the ED	1.4	19	35	17.4	15.8	3.8
Hospitalizations	1.9	15	15	12.4	9.2	4.8
Home-visits	N/A	147	N/A	17.4	18.2	3.2
