# Supplementary material for: The ecology of medical care on the westernmost remote island, Yonaguni Island, Japan: A cross-sectional study
Source: PLoS One. 2018 Jun 28;13(6):e0199871. doi: 10.1371/journal.pone.0199871 (PMC6023172; doi:10.1371/journal.pone.0199871)
Supplement: S1 File — (PDF) [file pone.0199871.s002.pdf]

Raw data

Visits											
Age	0-9	10-19	20-29	30-39	40-49	50-59	60-69	70-79	80-89	90>	Total
males	349	71	51	227	241	397	709	353	437	37	2872
females	311	103	72	296	265	302	714	540	571	151	3325
Total	660	174	123	523	506	699	1423	893	1008	188	6197

Visits				
Age	15<	15-70	70>	Total
male	415	1630	827	2872
females	412	1651	1262	3325
Total	827	3281	2089	6197
percent(%)	13.35	52.94	33.71	100

	Age	15<	15-70	70>	Total	Total
Referrals to the Outpatient Department	males	2	44	12	58	107
	females	5	27	17	49	
Secondry care clinic	males	0	5	1	6	12
	females	0	5	1	6	
Referrls to the Emergency Department	males	3	19	7	29	46
	females	0	3	14	17	
Admission after Referrls	males	3	22	9	34	57
	females	0	7	16	23	
Referrals to University	males	0	1	0	1	3
	females	0	2	0	2	
Over-night in the clinic	males	0	2	1	3	4
	females	0	0	1	1	

per month per 1000 inhabitants

	Visits			Referrals			Emergency			Hospitalizations			House-visits			Over-night		
Y.M. / Age	15<	15-70	70>	15<	15-70	70>	15<	15-70	70>	15<	15-70	70>	15<	15-70	70>	15<	15-70	70>
2015.7	51	243	198	1	10	11	0	1	2	0	3	6	2	1	0	0	0	0
2015.8	60	253	147	0	6	2	0	1	0	0	1	0	1	1	1	0	1	0
2015.9	78	269	175	0	4	4	0	0	1	0	0	1	1	0	0	0	0	0
2015.10	72	261	162	3	10	4	1	3	3	1	4	3	3	1	0	0	0	0
2015.11	52	271	174	0	11	4	0	2	3	0	2	3	3	0	0	0	1	0
2015.12	61	307	161	1	8	3	0	2	1	0	3	1	5	0	1	0	0	1
2016.1	71	282	198	0	9	5	0	1	3	0	2	3	2	1	1	0	0	1
2016.2	62	307	174	0	8	3	0	1	2	0	2	2	4	2	0	0	0	0
2016.3	77	293	198	1	9	6	1	2	4	1	2	4	4	0	0	0	0	0
2016.4	79	273	156	1	9	2	1	1	0	1	1	0	1	0	0	0	0	0
2016.5	83	259	174	1	11	5	0	3	0	0	3	0	2	0	0	0	0	0
2016.6	81	263	172	2	11	3	0	5	2	0	6	2	1	0	0	0	0	0
Total	827	3281	2089	10	106	52	3	22	21	3	29	25	29	6	3	0	2	2
